# Addressing the routine failure to clinically identify monogenic cases of common disease

**DOI:** 10.1186/s13073-022-01062-6

**Published:** 2022-06-07

**Authors:** Michael F. Murray, Muin J. Khoury, Noura S. Abul-Husn

**Affiliations:** 1 Yale Center for Genomic Health, Department of Genetics, Yale School of Medicine, 333 Cedar Street, New Haven, CT 06520 USA; 2 Office of Genomics and Precision Public Health, Office of Science, Centers for Disease Control and Prevention, 1600 Clifton Road, Atlanta, GA 30329 USA; 3 Institute for Genomic Health, Division of Genomic Medicine, Department of Medicine, Icahn School of Medicine at Mount Sinai, One Gustave L. Levy Place, Box 1041, New York, NY 10029 USA

## Abstract

Changes in medical practice are needed to improve the diagnosis of monogenic forms of selected common diseases. This article seeks to focus attention on the need for universal genetic testing in common diseases for which the recommended clinical management of patients with specific monogenic forms of disease diverges from standard management and has evidence for improved outcomes.

We review evidence from genomic screening of large patient cohorts, which has confirmed that important monogenic case identification failures are commonplace in routine clinical care. These case identification failures constitute *diagnostic misattributions*, where the care of individuals with monogenic disease defaults to the treatment plan offered to those with polygenic or non-genetic forms of the disease.

The number of identifiable and actionable monogenic forms of common diseases is increasing with time. Here, we provide six examples of common diseases for which universal genetic test implementation would drive improved care. We examine the evidence to support genetic testing for common diseases, and discuss barriers to widespread implementation. Finally, we propose recommendations for changes to genetic testing and care delivery aimed at reducing diagnostic misattributions, to serve as a starting point for further evaluation and development of evidence-based guidelines for implementation.

## Background

Adding a particular laboratory test to the standard management of a condition can be complex; however, the decision for routine inclusion can be reduced to two sets of issues: the evidence-base that supports testing, and implementation facilitators and barriers that impact the testing process. While laboratory tests to identify the underlying monogenic etiology of common diseases such as cancers and cardiovascular diseases have been available for decades [1, 2], genetic testing is not routinely incorporated into common disease management in most settings. As such, a failure to correctly ascribe a monogenic etiology to common disease occurs routinely [3–5]. This failure to test causes a “diagnostic misattribution,” and the consequence is that untested individuals with monogenic causes for their disease are managed in the same way as other cases who are assumed to have a polygenic or non-genetic etiology for their disease. We are writing to bring attention to diagnostic misattribution in common disease occurring when clinically useful genetic testing is available but not offered. This type of omission needs to be addressed in a manner that both meets the moment and lays an evidentiary foundation for moving forward.

The concept of diagnostic misattribution is not limited to genetics. It can be applied to other clinical scenarios where diagnostic studies are both available and useful but are not offered as part of the patient’s care management. As such, the decision to not offer the study leads to a lack of specificity in the diagnosis that results in sub-optimal care for some patients. Consider a hypothetical case of a health system deciding to limit brain imaging for patients presenting with acute stroke to those meeting a set of clinical criteria, despite the availability of imaging technology and evidence that the accurate identification of hemorrhagic strokes (15% of acute stroke cases) optimizes care and improves outcomes for acute stroke [6, 7]. Suppose the data show that these clinical criteria are insufficiently sensitive for identifying one in seven cases of hemorrhagic stroke, and therefore, these cases do not receive imaging. This hypothetical approach would undoubtedly lead to cases of hemorrhagic stroke being diagnostically misattributed to the more common type of stroke (i.e., ischemic stroke) and consequently result in missed opportunities for optimal care management [7].

When *BRCA1* genetic testing was first introduced in the mid-1990s, it was a new type of test to identify monogenic cases of breast cancer, and a positive result was informative but not associated with evidence-based management options [2]. The test was costly to perform and difficult to interpret, and additional research would still be required to determine how to assess novel variants encountered in such testing. For *BRCA1* and subsequently identified cancer predisposition syndromes, significant work was undertaken to identify individuals with the highest pre-test probability of a positive test result, and strategies were developed to limit testing to those individuals [8]. In addition, important ethical, legal, and social considerations were being addressed for the first time in relation to clinical genetic tests [9]. In this context, early implementation barriers, such as restricted insurance coverage for testing and an expectation for ordering providers to have genetics expertise, were in line with the state of knowledge at the time.

The current era is one where the consequences of diagnostic misattribution for *BRCA1* and other genes include missed opportunities for targeted evidence-based care of the patient [10]. We propose a new approach that starts with the question: Which clinical scenarios have reached a point where diagnostic attribution through genetic testing is actionable? In those instances where attribution prompts specific distinct care management steps, and where misattribution leads to missed opportunities for optimal care, health systems need to begin supporting reflexive genetic testing, i.e., genetic testing solely on the basis of the existing diagnosis.

The persistence of once justified implementation barriers to genetic testing in individuals with diagnosed disease now contributes to a recognized failure to routinely identify monogenic cases of common disease. We advocate for reflexive universal genetic testing to be considered for diseases for which monogenic case identification impacts care management and clinical outcomes for the affected patient. We propose piloting the initiation of changes to practice in the most compelling clinical scenarios where diagnostic misattribution interferes with optimal care. We include six examples (Table 1) for which the evidence for actionability is clear and consensus for universal testing is likely achievable, and discuss ways to address operational barriers to universal testing. Twelve discrete recommendations for addressing the barriers are proffered here as the types of steps needed to change medical practice (Table 2). We hope that this serves as a starting point to prompt discussion and debate aimed at defining consensus lists of actionable diseases that warrant reflexive genetic testing and addressing operational barriers to initiate important changes to care delivery in this arena.

**Table 1:** Common diseases in which a positive genetic test result is associated with changes to clinical management options for the patient

Common disease	Genes	Prevalence of “Disease Associated Variants” in general population	Prevalence of “Disease Associated Variants” in specific common disease	Potential targeted interventions associated with monogenic attribution		Guidelines related to diagnosis and/or management of the common disease and its monogenic forms	
				Pharmacologic therapy	Non-pharmacologic interventions	Professional organization(s)	Reflexive genetic testing recommended following diagnosis
Ovarian cancers^a^	*BRCA1* *BRCA2*	1:200 [5]	1:7 [11, 12]	Poly ADP ribose polymerase (PARP) inhibitors [13]	Bilateral mastectomy [14]	National Comprehensive Cancer Network (NCCN) [15] American Society of Clinical Oncology (ASCO) [16]	Yes
Breast cancer	*BRCA1* *BRCA2*	1:200 [5]	1:33 [17, 18]	PARP inhibitors [13]	Contralateral mastectomy Bilateral oophorectomy [19]	American Society of Breast Surgeons [20]^b^	Yes
Pancreatic cancer	*BRCA1* *BRCA2*	1:200 [5]	1:10 [21]	PARP inhibitors [22]	--	NCCN [15]	Yes
Colorectal cancer	*MLH1* *MSH2* *MSH6* *PMS2*	1:280 [23]	1:25 to 1:50 [24]	First-line therapy with pembrolizumab in advanced disease [25]	Transvaginal ultrasound, endometrial biopsy, hysterectomy [26]	Evaluation of Genomic Applications in Practice and Prevention (EGAPP) Working Group [27]	Yes^c^
Coronary artery disease	*LDLR* *APOB* *PCSK9*	1:250 [3]	1:20 [28, 29]	Lipid lowering for secondary prevention [combine statins with other lipid-lowering therapies as needed]	--	2018 AHA/ACC/AACVPR/AAPA/ABC/ACPM/ADA/AGS/APhA/ASPC/NLA/PCNA Guideline on the Management of Blood Cholesterol [30]	Not addressed
Hypertrophic cardiomyopathy	*MYH7* *MYBPC3* *TNNI3* *TNNT2* *TPM1* *MYL2* *MYL3* *ACTC1* HCM Mimics: [31] *PRKAG2* *LAMP2* *GAA* *GLA* *TTR*	1:200 to 1:400 [31, 32]	1:2 to 1:3 [33, 34]	If HCM mimic identified: enzyme replacement therapy (*GAA*, *GLA*) Antiarrhythmic drugs, ablation (*PRKAG2*) Transthyretin stabilizing or silencing drugs (*TTR*)	If HCM mimic identified: ICD implantation (*LAMP2*)	American College of Cardiology/American Heart Association Joint Committee on Clinical Practice Guidelines [35]	Yes

^a^“Ovarian cancers” include ovarian epithelial cancer, fallopian tube cancer, and primary peritoneal cancer - www.cancer.gov/types/ovarian/patient/ovarian-epithelial-treatment-pdq

^b^While the American Society of Breast Surgeons has recommended routine testing, the NCCN has not recommended reflexive testing

^c^In 2009, EGAPP noted that there was sufficient evidence to recommend offering genetic testing for Lynch syndrome to individuals with newly diagnosed colorectal cancer to reduce morbidity and mortality in relatives. This group has not revisited this topic since 2009. There is recent evidence for benefit to the patient in the form of FDA-approved targeted therapies [25], and this information was not available to EGAPP when they addressed genetic testing

**Table 2:** Implementation changes to consider in specific common diseases where actionable attribution can be revealed with a specific genetic test

Category	Item number	Recommended for consideration
Criteria for ordering monogenic tests in the diagnosed patient	*1*	Re-evaluation of the risks and benefits of employing genetic testing criteria checklists aimed at limiting who is offered testing to identify monogenic causes of common diseases
	*2*	Expansion of the ovarian cancer model that prompts reflexive genetic testing for all individuals with a given specific common disease diagnosis
Reflexive test offerings	*3*	Creation and maintenance of a list of common disease diagnoses and the specific reflexive gene tests they prompt by an authoritative group with credibility and standing across the healthcare community
	*4*	Creation of reflexive genetic testing panels that are designed to offer only those genes supported by clear evidence of clinical actionability for the diagnosed patient
Pre-test interaction	*5*	The association of reflexive genetic test implementation with approaches that assure equitable access in historically underserved populations
	*6*	Development of a standardized clinical consent process for reflexive genetic tests that can easily be completed by any competent healthcare professional
	*7*	Development of best clinical practices that offer reflexive genetic testing in a manner that does not require the involvement of individuals with advanced genetics training or expertise
	*8*	Offering reflexive genetic tests to patients with certain specific diagnoses without the expectation or requirement of a detailed family history ascertainment as part of pre-test discussion
Cost coverage	*9*	Re-evaluation of policies and practices that can (or do) result in a denial of coverage for reflexive genetic tests that seek to identify monogenic causes of common disease associated with actionable attribution
Test reporting	*10*	Attention to making the actionable results on laboratory reports clear to any competent provider, even a first-time user
	*11*	Clear communication from the testing laboratory of their plan and commitment to variant re-analysis and results updating so that the non-expert end-user does not need to worry about currently uninterpretable information (i.e., variants of unknown significance)
Result disclosure	*12*	Establishment of a defined standard of care for patients surrounding results on a reflexive genetic test in common disease

## Evolving landscape of monogenic causes of common diseases

Recently published examples from large DNA-based population screening programs within health systems have unambiguously demonstrated that [1] currently endorsed medical and family history-based screening strategies are not sufficiently sensitive to identify all individuals with monogenic disease-associated variants [5, 36, 37] and [2] patients with monogenic causes of common disease are routinely cared for without genetic testing [3–5]. In two reports, approximately 10% of screen-positive patients first found out about their disease-associated variant through the population screening program even though they had a standing diagnosis of the common disease in question (breast cancer, ovarian cancer, colorectal cancer, or coronary artery disease), because clinical genetic testing had not been previously performed [4, 5, 38].

In aggregate, an estimated 1–2% of the general adult population may have a readily identifiable genetic variant conferring a significantly elevated risk for cancer or cardiovascular disease [39]. In adults with specific diagnoses, the frequency of underlying monogenic disease-associated variants can be significantly enriched (see Table 1). For example, the population prevalence of monogenic familial hypercholesterolemia in the USA is 0.4% [3], but its prevalence in patients with acute coronary syndrome (ACS) is 4.7%, and in patients with ACS under 60 years is 7.4% [28, 29]. Hypertrophic cardiomyopathy (HCM), which affects up to 1 in 200 individuals in the USA [32], has an identifiable monogenic cause in 30–60% of cases, including a subset of patients with treatable metabolic or infiltrative diseases that mimic HCM [34, 35]. Approximately 3% of individuals with colorectal cancer have a mismatch repair gene variant associated with Lynch syndrome [24].

In the case of ovarian cancer, a 15% prevalence of disease-associated variants in *BRCA1* or *BRCA2* (*BRCA1/2*) has been reported in multiple studies [11, 12]. A recent study that captured population-based data in Georgia and California found that genetic testing rates associated with ovarian cancer diagnosis were only 30% [40]. Guidelines are in place from the National Comprehensive Cancer Network (NCCN), the American Society of Clinical Oncology, and other professional societies to offer reflexive testing of *BRCA1/2* and other genes in all diagnosed cases of ovarian cancer, with specific changes to care when testing is positive [15, 16, 41]. Focused efforts within individual institutions have demonstrated the potential to drive genetic testing rates in ovarian cancer to > 80% in response to such guidelines [42–44].

Genetic testing to accurately diagnose monogenic disease has the power to change care options for patients with ovarian cancer and other common diagnoses (see Table 1). There is also the potential for secondary benefits outside of direct disease management, such as better-informed prognoses, improved long-term preventive screening strategies, and targeted cascade testing of at-risk family members [45]. Primary disease management benefits for monogenic causes of common disease could be extended to more individuals if genetic testing was routinized in selected clinical scenarios. We recognize that, in order to achieve this, there need to be standardized definitions of actionable attribution so that the many stakeholders (e.g., clinical experts, clinical laboratories, payers, and patients) can reach a consensus. Such stakeholders consensus building will be fundamental to the evidence-based processes needed to create the list of common disease diagnoses and reflexive gene tests recommended in item 3 of Table 2. For those clinical scenarios achieving broad agreement, it will be crucial to address access barriers and disparities in genetic test implementation by establishing clear practice standards to support genetic testing of as many patients as possible [27].

Despite an increasing appreciation for identifiable monogenic forms of common diseases, genetic testing for these is not commonplace, even when guidelines for universal testing exist. The next sections will focus on the growing evidence base to support universal genetic testing for certain common diseases.

## Evidentiary foundation to genetic testing in common diseases

The evaluation of any genetic test in the context of a common disease includes an assessment of its analytic validity, clinical validity, and clinical utility, as well as associated ethical, legal, and societal implications [46]. While analytic validity (technical test performance) and clinical validity (sensitivity, specificity, and predictive value) have been established for many genetic tests, clinical utility may be less certain [47]. The clinical validity of genetic testing refers to its ability to accurately and reliably diagnose monogenic causes of the condition. As importantly, the clinical utility of genetic testing refers to its ability to inform clinical decision-making and improve health outcomes for patients and relatives [48].

For certain types of cancer, it is well established that genetic testing can reliably identify the monogenic etiology, which in turn provides prognostic information and informs systemic therapy selection [49, 50] and surgical decision-making [14, 19]. The identified genetic variant serves as a biomarker of future disease risk, providing an opportunity to implement additional screening and risk-reducing measures in order to prevent or make an earlier diagnosis of a second primary cancer. This is particularly important considering that, in a recent study of over 2000 patients with cancer, 11% had a germline disease-associated variant identified only after presenting with a second primary cancer [51]. The same study found that over 30% of patients with diverse cancer types harbored disease-associated variants, most of which were potentially actionable based on management guidelines, published expert opinion, FDA-approved precision therapy labels, or clinical trial eligibility criteria [51].

The clinical utility of routine genetic testing in non-cancer conditions is also emerging. In a recent study, almost 10% of adults with chronic kidney disease were found to have a disease-associated variant, the majority of which had implications for clinical management, such as disease reclassification and a search for extra-renal syndromic disease (e.g., hearing impairment in association with pathogenic variants in *COL4A5*) [52]. In patients with hyperlipidemia or ACS, identification of those with familial hypercholesterolemia enables early and aggressive cholesterol lowering to prevent or delay cardiovascular events [53, 54]. The identification of forms of cardiomyopathy due to infiltrative or metabolic diseases as opposed to primary muscle disease (also known as HCM mimics or HCM phenocopies) through genetic testing enables their targeted management (Table 1) [31]. One of these, cardiac amyloidosis, is now recognized as a frequently missed diagnosis in African Americans with heart failure [55, 56]. With the availability of new FDA-approved targeted therapies that delay amyloidosis progression [57], genetic testing for *TTR* V142I, a founder variant present in 3–4% of African Americans, could improve outcomes for this disease [58–60]. Importantly, although the optimization of clinical care is only enabled by the accurate identification of such monogenic forms of common disease, the vast majority of these go unrecognized in routine clinical care today.

Many other common diseases have monogenic subsets, for which genetic testing is not routinely applied. Examples include monogenic forms of diabetes, atrial fibrillation, and dementia. Decisions about which common diseases are ready for strategy change to universal genetic testing will require processes to define evidence-based criteria for actionability related to monogenic forms of those diseases and to re-evaluate these over time. We believe it will take a multi-stakeholder group with credibility and standing to create such lists (see Table 2).

## Implementation barriers to genetic testing in common diseases

Clinical genetic testing has been widely available for many years and can be readily used to identify common disease cases with monogenic etiology. However, only a small proportion of cases obtain testing. Even with *BRCA1/2*-associated breast cancer, which is likely the best-known instance of a monogenic cause of common disease, the majority of cases are missed due to a lack of genetic testing [61]. Furthermore, racial and ethnic disparities in *BRCA1/2* testing among breast cancer survivors are well documented [62–64] and contribute to health inequities in genomic medicine [65].

Barriers to genetic testing are multifaceted. Arguably, the most significant obstacle to obtaining genetic testing for patients with common disease diagnoses is the real or perceived requirement for a healthcare professional with specific genetics expertise to be involved. Requirements for consultation with a certified genetic counselor or medical geneticist prior to genetic testing have been implemented by some health insurers [66], with the intent to guide appropriate genetic testing in the context of growing demand. Professional societies have argued that such requirements impose barriers to timely diagnosis and unnecessarily restrict the scope of practice of non-geneticist physicians [67]. Straightforward testing guidelines for common diseases in which genetic testing is indicated, as well as establishing pre- and post-test standards of care could both remove implementation barriers and address any concerns about adequate support of patients and providers. Given the relative scarcity of medical geneticists and genetic counselors [68], the traditional referral model for genetic testing can impede access to care, result in poor patient compliance, and further exacerbate health disparities [66, 69–71].

On the other hand, practical implementation of genetic testing in routine care may be hindered by the complexity of genetic test offerings and guidelines. Current guidelines for genetic testing include criteria considering the number of affected relatives and their ages at diagnosis. While a detailed family history can inform pre-test probability, for a patient presenting with an existing diagnosis, it cannot determine whether genetic testing will benefit the patient’s disease management [61]. The risks and benefits of extensive pre-test counseling and family history ascertainment should be re-evaluated in this context.

The landscape of genetic testing is rapidly evolving, with multiple test modalities and test options commercially available. Multigene panel testing has largely replaced single gene testing in cancer and other disease areas [72]. While this approach can increase the sensitivity to detect pathogenic variants, it also creates challenges to routine testing. Large multigene panels often include lower-penetrance genes or genes with less-established disease associations and undefined actionability, and increase the number of variants of uncertain significance (VUS) detected, particularly in non-European descent individuals [72]. The creation of standardized genetic testing panels designed to offer only genes supported by clear evidence of clinical actionability should be considered. The practice of routinely including VUS in test reports should also be re-evaluated, since it appears to add complexity and cost without a beneficial influence on the care provided by non-expert clinicians [73]. The misinterpretation of VUS by non-genetics providers can result in inappropriate management [73, 74]. While clinicians with genetics expertise may desire VUS reporting to enable further investigation over time for evidence of pathogenicity, these experts do not represent the majority of caregivers who will be receiving genetic test reports.

In order to enable routine genetic testing for certain common diseases, measures will need to be taken to simplify the testing process and result interpretation, making it akin to other types of clinical tests. The approach to testing needs to be streamlined so that non-genetics providers can routinely offer testing. Reflexive testing should include only genes recommended based on specific clinical utility [75, 76]. In addition, approaches to ensure equitable access to genetic testing in historically underserved populations are needed. Considerations for how to address some of the significant operational barriers to universal genetic testing in the USA are summarized in Table 2. Commonalities and differences in these barriers across countries will need to be further explored and addressed.

## Implications for diagnostic misattribution in clinical practice

If a genetic test result does not drive specific management considerations, then it could be argued that the failure to identify a genetic basis for disease through genetic testing is a benign lack of diagnostic specificity. For example, this lack of specificity could be invoked at the present time relative to polygenic risk scores for breast cancer or coronary artery disease, which are associated with statistically increased disease risk but are not yet linked to evidence-based management recommendations [77]. However, the lack of genetic testing when a positive result would prompt clear actionable clinical care is another matter. Failure to test in this instance appears to be at the threshold for a “diagnostic error,” which was defined in the recent framework from the National Academies as “the failure to establish an accurate and timely explanation of the patient’s health problem” [78]. We would caution that diagnostic misattribution that occurs when monogenic disease is managed as polygenic or non-genetic disease, resulting in missed opportunities for care, could before long come to be interpreted as a diagnostic error.

The need for improved implementation of genetic testing is clear with the case of *BRCA1*, where testing was first offered for clinical use in 1996 and yet monogenic case identification failures in the untested remain commonplace. Results of a recent study demonstrated that if existing testing criteria were applied to a universally tested cohort of women with breast cancer, then 9.8% of the individuals with *BRCA1* and 16.1% of the individuals with *BRCA2* disease-associated variants would not have had testing recommended [79]. At this point, 25 years after the introduction of the genetic test, the rationale to support the continued use of insufficiently sensitive criteria-based strategies to determine who among diagnosed individuals is offered a genetic test in this circumstance is unclear.

Given the ethical, legal, and social issues associated with genetic testing, the debate about whether and how genetic testing should be managed as compared to other laboratory tests remains an important one [80]. Nonetheless, it is time to re-evaluate some of the current practices that foster diagnostic misattribution, including elements designed to offer testing only to individuals with a discernible high pre-test probability. This strategy has proven difficult to routinely incorporate and insufficiently sensitive, both of which limit appropriate monogenic attribution. Routine genetic testing will require changes to the current workflows for healthcare providers seeking to order a genetic test. In order to equitably extend the benefits associated with genetic testing, it cannot be contingent on the capacity of a small specialized provider workforce. An improved workflow for genetic testing will require behavioral adjustments for healthcare providers, genetic testing laboratories, and payers to become more like other laboratory testing workflows. The proposed implementation strategies to address current obstacles to routine genetic testing (Table 2) may not be sufficient to support a major shift in medical practice to offer universal genetic testing. To maintain the trust of patients and providers, there will need to be adequate attention to data privacy, data security, and legal gaps related to the Genetic Information Non-discrimination Act (GINA) [81]. Ancillary changes that are needed include increased genetics and genomics education of providers and patients, improved integration of genetic test results into electronic health records, clinical decision support linking evidence-based recommendations with positive test results, improved information systems and analytics, support for cascade testing of at-risk relatives, and clear patient care navigation, including the designation of follow-up responsibility among providers for new aspects of routine care.

An evidence-based list of conditions recommended for reflexive genetic testing will need to be proposed, evaluated, and maintained, similarly to the list of conditions recommended for newborn screening panels [82]. A reasonable starting point for re-evaluation could be conditions from Table 1, for which genetic testing informs management and has established clinical utility; conditions beyond these will require evidence-based data collection. Additional studies will be needed to evaluate and refine alternative genetic test implementation strategies. Large-scale patient cohorts with adequate representation from diverse populations could accelerate our knowledge in this area. Such studies could be based on biobanks embedded in health systems, which offer opportunities to evaluate and implement reflexive genetic testing for proposed conditions with longitudinal follow-up of health-related outcomes [83].

The strategic vision of the National Human Genome Research Institute includes the bold prediction that by 2030 “the regular use of genomic information will have transitioned from boutique to mainstream in all clinical settings, making genomic testing as routine as complete blood counts” [84]. We note that ordering a complete blood count does not require a competent clinician to engage a hematologist, and suggest here that ordering a genetic test should not require a competent clinician to engage a genetics clinician. If we are to usher in genomic medicine in a manner that offers maximum benefit to patients, then we are obligated to define best practices that empower providers and equitably improve care. We anticipate that a continued failure to develop widely accepted approaches for addressing the current problem of diagnostic misattribution may bring us to a tipping point where forces acting on behalf of patients from outside of healthcare delivery, such as legislative bodies, malpractice liability claims, or increased reliance on direct-to-consumer testing, will prompt their own versions of change to address the current practice gap.

## Conclusions

Diagnostic misattribution is a potential category of diagnostic error and like other categories (e.g., delayed diagnosis, misdiagnosis, and over-diagnosis) is not limited to genomic medicine. As opposed to a misdiagnosis, diagnostic misattribution is a correct diagnosis that lacks specificity when it matters. In any area of medicine, misattributions that demand attention are those that limit the optimal care management of affected patients despite the ready availability of the technology to correct the misattribution.

Patients in every health system are currently missing opportunities for optimal genomic medicine-associated care due to diagnostic misattribution. Universal genetic testing following the diagnosis of selected common diseases will address the care gap that has been created when access to these readily available laboratory tests is either intentionally or unintentionally limited. Changes aimed at facilitating the reflexive use of genetic testing are needed, and this massive change to medical practice is unlikely to occur all at once or uniformly across healthcare. As has been demonstrated in the case of progress toward criteria-free genetic testing in ovarian cancer [42–44], improvements in diagnostic attribution will likely need to be modeled first at the individual healthcare system level prior to broad adoption.

Our opinion is that a top genomic medicine priority is to actively consider universal genetic testing for monogenic forms of common disease when identification of a monogenic etiology will drive evidence-based changes in care management and improved outcomes. This important addition of genetic testing to routine care delivery will need further evaluation and will need to be made as uncomplicated to order, interpret, and implement as other portions of the common disease work-up that follow initial disease diagnosis.

## Data Availability

Not applicable.
